# Acute and sub-acute oral toxicity of ethanol extract of *Cassia fistula* fruit in male rats

**Published:** 2019

**Authors:** Rizwana Abid, Riaz Mahmood

**Affiliations:** *Department of PG Studies and Research in Biotechnology and Bioinformatics, Jnanasahyadri, Kuvempu University, Shankaraghatta, Shivamogga, Karnataka, India*

**Keywords:** Cassia fistula ethanol extract, Toxicity, Histopathology, Haematology

## Abstract

**Objective:**

The plant *Cassia fistula* L. (Caesalpiniaceae) is traditionally used to treat heart diseases, abdominal pain and fever. The present study was aimed to investigate the toxic effects acute and sub-acute administration of ethanol extract of *C. fistula* fruit (CFE) in male Wistar rats.

**Materials and Methods:**

In acute toxicity, the effects of a single oral dose (1000, 3000 and 5000 mg/kg) of CFE have been determined. Animal behaviour and mortality were determined for up to 14 days. In sub-acute study, the effects of CFE in daily single oral administration at the doses of 250, 500 and 1000 mg/kg during 28 days were determined. The blood haematological and biochemical parameters, as well as the histopathological examination of the liver, heart and kidneys were studied.

**Results:**

In acute study, a single administration of the CFE up to a dose of 5000 mg/kg did not induce mortality. Thus, the LD_50_ of the CFE has been estimated higher than 5000 mg/kg. In sub-acute toxicity study, administration of CFE at the doses of 250, 500 and 1000 mg/kg to rats did not induce mortality. No significant differences were found in relative organ weight, and haematological and biochemical analyses in treated groups compared to control group. No noticeable histological changes were observed in organs of CFE-treated rats compared to controls.

**Conclusion:**

These results have shown that oral administration of *C. fistula* fruit did not produce any significant toxic effect in male rats. Hence, *C. fistula* fruit could be regarded as a safe natural product for therapeutic use.

## Introduction

Nature has been a great source for therapeutics for thousands of years and plant-based products continue to play a key role in the primary health care of about 80-85% of the world’s population (Dubey et al., 2004 ▶). Medicinal plants has increasing gained public and professionals acceptance due to progresses in the understanding of the mechanisms by which these plants positively influence health and quality of life (Huh and Staba, 1992 ▶). Medicinal plants are widely used for the treatment of a variety of diseases and assumed to be safe; however, some medicinal plants can potentially be harmful at excessive doses or can interact with modern drugs (Saad et al., 2006 ▶). According to the World Health Organization (WHO), there is an increasing public demand for research on bridging knowledge gaps about safety of medicinal plants (WHO, 2004 ▶).

*Cassia fistula *L. (Caesalpiniaceae), commonly known as Indian laburnum/Golden Shower is a medicinal plant of immense importance and it could be found in India, Pakistan, Egypt, Ghana, Zimbabwe, Thailand, Malaysia and Sri Lanka. It has been widely used in different types of traditional medicines including Ayurveda, Unani and Chinese for various ailments. *C. fistula* is rich in anthraquinones, flavonoids, flavan-3-ol, and β-sitosterol (Chauhan et al., 2011 ▶). The rural people of India consume the matured fruit pulp as a purgative and a treatment for heart diseases and abdominal pain (Kirtikar and Basu, 1975 ▶). Extracts of various parts of the plant have been reported to demonstrate a variety of pharmacological activities such as antioxidant, anti-inflammatory, hypolipidemic, anti-tumor, anti-diabetic, antimicrobial, hepatoprotective, anti-pyretic, anti-fertility, anti-ulcer, larvicidal, ovicidal and wound healing effects (Abid et al., 2014 ▶; Rahmani, 2015 ▶; Abid et al., 2016 ▶).

It is well-known that consumption of medicinal plants without evaluating their efficacy and safety profile can result in toxic effects that may affect different organs. The liver and kidneys are the first targets because they are involved in the metabolism and excretion of chemical compounds (Hodges and Minich, 2015 ▶). However, there is lack of experimental data on the toxicity of *C. fistula* fruit. Despite the widespread use of this plant, its oral toxicological profiles have not been studied. Therefore, the present study was undertaken to examine the possible toxic effect of ethanol extract of *C. fistula* fruit in male Wistar rats following a single oral administration as well as 28 consecutive day administrations. 

## Materials and Methods

**Chemicals**

Diagnostic test kits were purchased from Robonik (India) Pvt. Ltd. Mumbai, India. All other chemicals and solvents used were of analytical grade. 

**Plant material and preparation of the extract**

Fruits (ripen pods) of *C. fistula *were collected in May 2012 from the orchard of Bioscience complex, Kuvempu University, Shivamogga, Karnataka, India. The plant was identified by a taxonomist, Department of studies in Botany, Kuvempu University, by comparing it with the authenticated specimen deposited at the Kuvempu University Herberia (Voucher No. KU/BS/MG 008).

Fruits were shade dried at room temperature and pulverized mechanically to a coarse powder. The powdered material was defatted using petroleum ether in Soxhlet extractor. Further, the defatted material (500 g) was subjected to sequential hot extraction process with chloroform (2 L, 45 °C, ≈15 cycles) followed by ethanol (2 L, 50 °C, ≈15-17 cycles). The ethanol extract was concentrated to dryness under reduced pressure in a rotary evaporator (Buchi, Flawil, Switzerland). The yield of *C. fistula* ethanol extract was 22.09%, (w/w). The extract was labelled as CFE and stored in desiccator to avoid oxidation until further use.

**Animals**

Male Wistar albino rats of 9 weeks old and 150-180 g weight, were used for the acute 14-day and sub-acute 28-day toxicological studies. The rats were obtained from inbred disease-free animal house, National Institute of Pharmacy, Shivamogga, Karnataka, India and acclimatized to laboratory conditions for a week prior to the experimentation. Six rats per cage were housed and kept in a controlled environment at an ambient temperature of 25±2 °C, relative humidity of 45-50% with 12 hr/12 hr light and dark cycles. Animals were allowed for standard laboratory diet (Hindustan Lever Ltd., Bangalore, India) and water *ad libitum* during the experiment. The study received approval from the Institutional Ethical Committee (Registration No. NCP/IAEC/CL/204/01/2013-14).

**Acute toxicity study**

Acute toxicity of CFE was evaluated as per the Organization for Economic Co-operation and Development (OECD) guideline number 423, using male Wistar albino rats (OECD, 2001 ▶). Experimental animals were divided into 4 groups of 6 animals. They were fasted for 12 hr prior to the experiment with free access to water. The control group was treated with distilled water, and the other 3 groups were orally administered with a single dose of CFE 1000, 3000 and 5000 mg/kg. Treated animals were continuously observed for any changes in general behaviour, food and water consumption, and mortality for 30 min and then intermittently followed for 4 to 24 hr after administration of CFE. The rats were further observed for up to 14 days following the treatment. At the end of the treatment (day 15), the animals were sacrificed for the macroscopic observation of internal organs. 

**Sub-acute toxicity study**

This study was performed as per the OECD guidelines 407 (OECD, 2008 ▶). Animals were divided into 4 groups of 6 rats. The control group was treated with distilled water, and the other 3 groups were daily treated by oral administration of CFE at different doses of 250, 500, 1000 mg/kg for 28 days. All the experimental animals were observed daily for any abnormal clinical signs, body weight change and mortality for 28 days. On day 28, the animals were subjected to overnight fasting and later sacrificed under mild anaesthesia. Blood samples were collected through cardiac puncture into non-heparinized tubes for biochemical analyses and into EDTA tubes for haematological analyses. The liver, heart and kidneys were collected for histopathological examinations. 

**Haematological analysis**

In haematological studies, red blood cells (RBC), white blood cells (WBC), haemoglobin, mean corpuscular volume (MCV), mean corpuscular haemoglobin (MCH) and mean corpuscular haemoglobin concentration (MCHC) were determined using an automatic haematology analyzer (Sysmex, Kobe, Japan). 

**Biochemical analysis**

For biochemical analysis, blood without additive was centrifuged at 4000 rpm at 4 °C for 10 min to collect the serum and tests were done using biochemical analyzer Robonik (Robonik India Pvt. Ltd., New Mumbai). Biochemical studies were carried out for liver function (in terms of aspartate transaminase (AST), alanine transaminase (ALT), alkaline phosphatase (ALP), and total bilirubin levels), kidney function (urea and creatinine levels), heart function (creatine kinase-MB level) and other biochemical parameters (total protein, glucose and total cholesterol levels). Relative organ weights (for the liver, kidneys, heart, lungs and spleen) were recorded in order to detect possible toxic effect of CFE at the macroscopic level. Relative organ weight was calculated using the formula:

Relative organ weight = [Absolute organ weight (g) / Body weight of rat on sacrifice day (g)] × 100 

**Histopathological examination**

For histopathological studies, the liver, heart and kidney were dissected out from the animals of each group and washed with normal saline and immediately fixed in 10% formalin. The organs were dehydrated in gradual grades of ethanol (50–100%), cleared in xylene and embedded in paraffin. Sections of 3- 5 µm thickness were prepared using a rotary microtome, processed in alcohol- xylene series, stained with haematoxylin and eosin (H–E) dye and observed under microscope (Nikon Eclipse 80i, Tokyo, Japan).

**Statistical analysis**

All the experiments were performed in triplicate and results were presented as mean±SEM. The statistical analysis was carried out using one way ANOVA followed by Dunnett’s multiple comparison test. The differences in values of p<0.05 or p<0.01 were considered statistically significant. Data was computed for statistical analysis by using GraphPad prism 5. 

## Results

**Acute oral toxicity study**

In the acute toxicity study, oral administration of a single dose up to 5000 mg/kg did not exhibit any mortality nor signs of toxicity during the observational period of 14 days. No significant changes in the body weight gains were detected. Therefore, the approximate lethal dose (LD_50_) of CFE was estimated to be higher than 5000 mg/kg.

**Sub-acute oral toxicity study**

**General behaviour and mortality**

Administration of CFE for 28 consecutive days did not induce behavioural changes at any time point in treated rats compared to the control group. No mortality was observed during the experimental period. 

**Body weight and relative organ weight**

**Figure 1:**
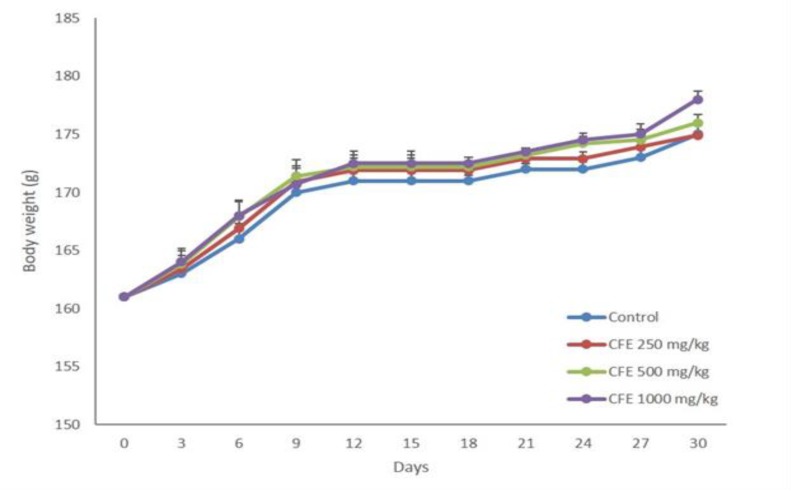
Effect of ethanol extract of *C. fistula* (250, 500 and 1000 mg/kg) on mean body weight in male rats in sub-acute toxicity study. Values are means±SEM of six rats

There was a progressive increase in the body weights of rats during the sub-acute treatment (Figure 1). Daily administration of CFE at different doses (250, 500 and 1000 mg/kg) for 28 days did not result in any significant change in the mean body weight of CFE-treated groups compared to the control. There was no significant effect on relative organ weight in treated and control groups (Table 1). 

**Table 1:** Effect of sub-acute administration of *C. fistula* extract on the relative weight of organs (n=6)

**Organ**	**Relative weight of organs (%)**			
	**Control**	**Extract dose**		
		**250mg/kg**	**500mg/kg**	**1000mg/kg**
**Liver**	3.57±0.32	4.14±0.38	3.99±0.11	4.04±0.23
**Heart**	0.33±0.01	0.34±0.02	0.33±0.01	0.34±0.02
**Lungs**	0.68±0.07	0.79±0.09	0.66±0.01	0.70±0.07
**Kidney**	0.77±0.03	0.80±0.07	0.78±0.03	0.79±0.06
**Spleen**	0.42±0.02	0.51±0.05	0.56±0.03	0.56±0.11

Values are expressed as mean±SEM.

No significant differences were observed.

**Haematological parameters**

The effects of sub-acute administration of CFE on haematological parameters are illustrated in Table 2. Daily administration of CFE for 28 days did not cause any significant change in haematological parameters tested [i.e. red blood cells (RBC), white blood cells (WBC), haemoglobin, mean corpuscular volume (MCV), mean corpuscular haemoglobin (MCH) and mean corpuscular haemoglobin concentration (MCHC)] at all the three doses of 250, 500 and 1000 mg/kg when compared to normal control. In other words, all the haematological values in all treated groups were remained within the physiological range after the experimental period. 

**Table 2:** Effect of sub-acute administration of *C. fistula* extract on haematological parameters in male rats (n=6)

**Parameters**	**Control**	**Extract dose**		
		**250mg/kg**	**500mg/kg**	**1000mg/kg**
**RBC** **(10** ^6^ **/µL)**	9.12±0.5	9.15±0.5	9.14±0.5	9.11±0.5
**WBC** **(10** ^3^ **/µL)**	11.76±0.05	12.11±0.01	11.24±0.49	11.13±0.12
**Haemoglobin** **(g/dL)**	11.39±0.03	11.59±0.06	11.62±0.13	11.65±0.50
**MCV** **(fL)**	52.06±0.44	53.64±0.03	54.53±0.02	53.83±1.83
**MCH** **(pg)**	17.23±0.02	17.28±0.02	17.49±0.01	17.26±0.01
**MCHC** **(g/dL)**	32.14±0.01	32.24±0.02	32.21±0.01	32.15±0.05

Values are expressed as mean±SEM.

No significant differences were observed.

**Biochemical parameters**

The effects of sub-acute administration of CFE on biochemical parameters are depicted in Table 3. Repeated administration of CFE for 28 days at 500 mg/kg in rats induced a slight decrease in ALT level when compared to control group. All other parameters such as hepatic biomarkers (AST, ALP and total bilirubin), kidney function markers (urea and creatinine), cardiac function marker (creatine kinase-MB) and other biochemical parameters (total protein, glucose, and total cholesterol) showed normal levels. 

**Table 3:** Effect of sub-acute administration of *C. fistula* extract on biochemical parameters in male rats (n=6)

**Parameters**	**Control**	**Extract dose**		
		**250mg/kg**	**500mg/kg**	**1000mg/kg**
**ALT** **(IU/L)**	247.8±3.67	218.2±13.08	211.8±32.07*	213.1±8.55
**AST** **(IU/L)**	205.9±3.39	197.5±2.33	226.8±11.05	228.5±13.71
**ALP** **(IU/L)**	142.7±2.9	162.9±12.45	150.0±4.36	147.6±4.66
**Total bilirubin** **(mg/dL)**	0.51±0.01	0.46±0.05	0.52±0.01	0.55±0.01
**Total protein** **(g/dL)**	6.81±0.15	7.02±0.45	6.93±0.01	6.95±0.17
**Glucose** **(mg/dL)**	87.83±0.7	88.73±0.61	88.62±0.15	88.38±0.62
**Urea** **(mg/dL)**	14.05±0.05	15.55±0.05	15.55±0.25	13.85±0.05
**Creatinine** **(mg/dL)**	0.59±0.01	0.59±0.05	0.59±0.02	0.58±0.03
**Total cholesterol** **(mg/dL)**	93.56±1.74	108.5±6.55	103.6±0.55	95.42±1.42
**CK-MB** **(IU/L)**	297.9±2.77	274.9±7.11	263.3±6.89	253.4±6.26

Values are expressed as mean±SEM.

* p<0.05 shows significant difference when compared to corresponding control.

**Figure 2:**
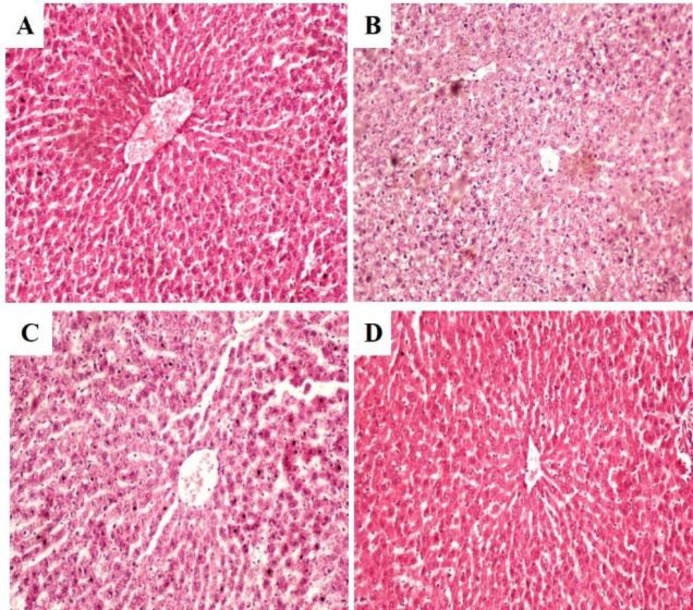
Photomicrographs of liver sections of rats administered with ethanol extract of *C. fistula* for 28 days (haematoxylin–eosin): (A) Normal control rats, showing normal hepatic histology; (B) Rats treated with 250 mg/kg of CFE, showing normal hepatic architecture; (C) Rats treated with 500 mg/kg of CFE, showing more or less normal hepatic architecture and (D) Rats treated with 1000 mg/kg of CFE, showing normal hepatic histology (magnification 40X).

**Histopathological examination**

Histopathological examinations of liver section in rats treated with CFE are shown in Figure 2. Microscopic observation of liver sections of normal control showed healthy hepatic cells without fatty lobulation, well-preserved cytoplasm, prominent nucleus and intact central vein (Figure 2A). Rats administered with CFE 250, 500 and 1000 mg/kg showed normal liver architecture with no necrosis (Figure 2B-2D). 

**Figure 3:**
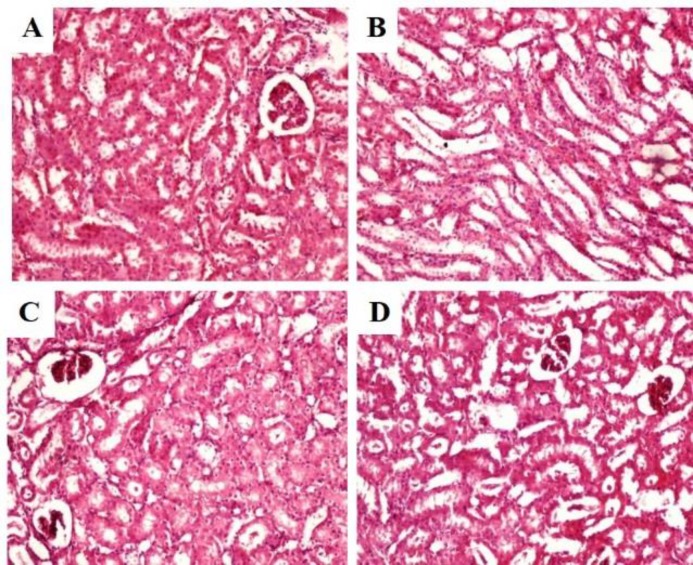
Photomicrographs of kidney sections of rats administered with ethanol extract of *C. fistula* for 28 days (haematoxylin–eosin): (A) Normal control rats, showing intact glameli and tubules; (B) Rats treated with 250 mg/kg of CFE, showing normal histology; (C) Rats treated with 500 mg/kg of CFE, showing normal histology and (D) Rats treated with 1000 mg/kg of CFE, showing normal histology (magnification 40X)

The results of histopathological examination of kidney sections from rats treated with CFE, are shown in Figure 3. The kidneys from rats administered with distilled water (control group) demonstrated intact glomeruli and tubules (Figure 3A). Rats administered with CFE at 250, 500 and 1000 mg/kg showed a normal architecture structure similar to the control group (Figure 3B-3D).

**Figure 4:**
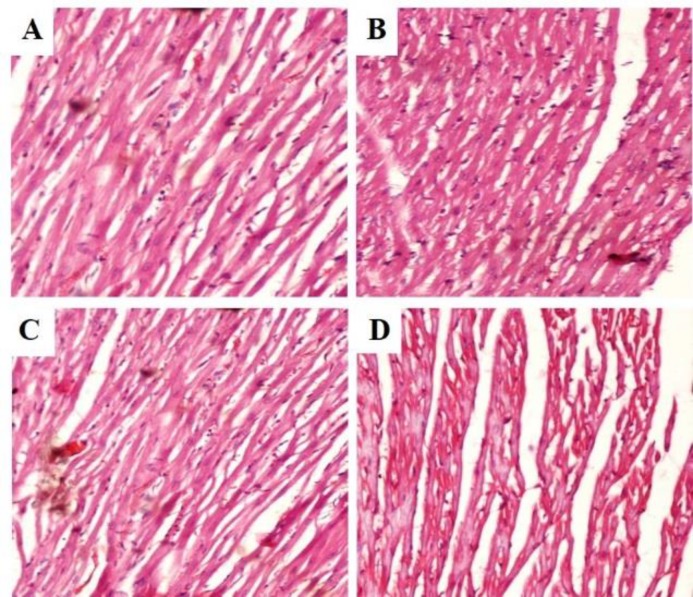
Photomicrographs of heart sections of rats administered with ethanol extract of *C. fistula* for 28 days (haematoxylin–eosin): (A) Normal control rats, showing normal myofibrillar structure with striations and branched appearance; (B) Rats treated with 250 mg/kg of CFE, showing normal cardiac architecture; (C) Rats treated with 500 mg/kg of CFE, showing normal cardiac histology and (D) Rats treated with 1000 mg/kg of CFE, showing normal cardiac architecture (magnification 40X)

Histopathological examination of heart sections from rats treated with CFE are shown in Figure 4. The histological analysis of hearts of normal control and CFE-treated rats showed a normal myofibrillar structure with striations, branched appearance that is a normal cardiac histology (Figure 4A-4D).

## Discussion

Though *C. fistula* is extensively used in the traditional medicine for the treatment of various ailments including liver conditions and heart diseases, there is little scientific and clinical data on the effectiveness and the safety of *C. fistula* fruit extract. To investigate the plant’s safety profile, acute and sub-acute toxicity evaluations of ethanol extract of *C. fistula *were performed in male rats. Previous studies revealed that extracts of this plant exhibit a variety of pharmacological activities such as antioxidant, anti-inflammatory, hypolipidemic, anti-tumor, anti-diabetic, antimicrobial, hepatoprotective, anti-pyretic, anti-fertility, anti-ulcer and wound healing effects (Abid et al., 2014 ▶; Rahmani, 2015 ▶; Abid et al., 2016 ▶). 

In addition, phytochemical constituents present in different parts of *C. fistula* are anthraquinone, flavanol and flavonol derivatives such as rhein, chrysophanol, emodin, physcion, fistulic acid, sennoside A and B, (+) catechin, kaempferol, (-) epicatechin, procyanidin B2, (-) epiafzelechin, (+) epiafzelechin, rhamnetin-3-O-gentiobioside; flavonoids and proanthocynidins (Bahorun et al., 2005 ▶; Chauhan et al., 2011 ▶). Bahorun et al. (2005) ▶ and Abid et al. (2014) ▶ showed that flavonoids such as kaempferol, catechin, epicatechin, procyanidin B2, epiafzelechin, quercetin, myricetin, and rutin are present in the fruit of *C. fistula*. These metabolites have important pharmacological activities but they can also induce toxicological effects. Some of these compounds are known to possess haematotoxic and hepatotoxic properties, and may provoke depressive activity on the central nervous system, and cause mutagenesis and carcinogenesis (Wiarm et al., 2005 ▶; Michalowicz and Duda, 2007 ▶). Therefore, evaluation of *C. fistula* toxicity is indispensable. 

In the present study, several parameters were assessed after *in vivo *acute and sub-acute administration of ethanol extract of *C. fistula* fruit. Mortality is an important criterion in toxicological assessments (Asare et al., 2012 ▶); both acute and sub-acute administration of the extract did not induce a significant mortality. In the acute toxicity study, administration of a single dose up to 5000 mg/kg of CFE did not exhibit any signs of toxicity nor mortality during the entire observation period. Therefore, the approximate lethal dose (LD_50_) of *C. fistula* fruit extract is estimated to be greater than 5000 mg/kg. According to the Globally Harmonized System (GHS) of Classification and Labelling of Chemicals, the substances with oral LD_50_ values of >5000 mg/kg are considered relatively safe following acute exposure (United Nations, 2011 ▶).

Determination of weight gain or relative organs’ weight is important to distinguish possible organ damages by exposure to toxic substances. The weight of damaged organ would be altered depending on the extent of toxicity and to the ratio of body weight (Rosidah et al., 2009 ▶). In the sub-acute toxicity study, CFE administered up to 1000 mg/kg, did not cause any change in clinical signs, mortality nor morbidity. In addition, it did not elicit any deleterious effect with regard to relative organs’ weight compared to the control animals.

The hematopoietic system which is one of the most vulnerable targets of toxic substances, reside in the bone marrow where the production of red blood cell occurs (Birbrair and Frenette, 2016 ▶). This system is an important index of physiological and pathological states in both men and animals (Mukinda and Syce, 2007 ▶). In this study, sub-acute administration of CFE did not cause significant changes in the haematological profile of rats that received different doses of CFE when compared with control, suggesting that CFE is probably not toxic to the blood system.

Assessment of liver function is essential in evaluating the toxicity of drugs and plant extracts (Clarke and Clarke, 1977 ▶). AST, ALT, ALP, total serum protein and total bilirubin concentration in serum are the most common clinical biomarkers of liver condition (Al-Mamary et al., 2002 ▶; Mayne, 1996 ▶). In the present study, no significant alterations were observed in serum levels of AST, ALT, ALP, total protein and total bilirubin in sub-acute CFE fed groups. The present study found that, administration of CFE up to a dose of 1000 mg/kg, does not exert any adverse effects on the liver. Similarly, the histological examination of the liver also did not show any changes in the tissue, confirming that extract administration is relatively safe for the liver.

The kidneys are considered frequent targets of toxicity (Dekant and Vamvakas, 1996 ▶). CFE-fed groups showed insignificant differences in the level of urea and creatinine. It indicates that CFE might not affect the normal renal function following sub-acute exposure when compared to the control group. In addition, no significant change was observed in the blood glucose level in sub-acute CFE-fed groups. Similarly, histological analysis showed no alterations in the tissue architecture of the organ following sub-acute administration of CFE.

High cholesterol levels could be explained by the stimulation of lipid anabolism in hepatocytes under the action of the extract or an exogenous supply of fatty compounds contained in the extract (Jaouhari et al., 1999 ▶). Administration of CFE at all tested doses had no effect on total cholesterol level. CK-MB was determined as an important cardiac marker enzyme; higher levels of this enzyme are of relevance to myocardial infarction (Asare et al., 2011 ▶). CFE-fed groups did not show any change in the level of CK-MB when compared to control group. In addition, histological evaluation of the heart did not show tissue changes of any kind in sub-acute treated groups. 

In conclusion, the oral doses of *C. fistula* fruit extract can be considered safe as they did not exhibit any lethality nor adverse effects in the acute and sub-acute toxicity studies in male rats. The present study provides supportive data on the use of *C. fistula* in traditional medicine and could lend credence for its medicinal use. Chronic toxicity, mutagenicity and carcinogenicity evaluations should be performed to have a better understanding of the plant’s safety profile.
